# Photooxidation Behavior of a LDPE/Clay Nanocomposite Monitored through Creep Measurements

**DOI:** 10.3390/polym9080308

**Published:** 2017-07-26

**Authors:** Francesco Paolo La Mantia, Mario Biondo, Maria Chiara Mistretta, Fiorenza Sutera, Marco Morreale

**Affiliations:** 1 Dipartimento di Ingegneria Civile, Ambientale, Aerospaziale, dei Materiali, Università di Palermo, Viale delle Scienze, 90128 Palermo, Italy; MarioBiondo88@hotmail.it (M.B.); mariachiara.mistretta@unipa.it (M.C.M.); fiorenza.sutera@unipa.it (F.S.); 2 Facoltà di Ingegneria e Architettura, Università di Enna “Kore”, Cittadella Universitaria, 94100 Enna, Italy; marco.morreale@unikore.it

**Keywords:** creep, photooxidation, nanocomposites

## Abstract

Creep behavior of polymer nanocomposites has not been extensively investigated so far, especially when its effects are combined with those due to photooxidation, which are usually studied in completely independent ways. In this work, the photooxidation behavior of a low density polyethylene/organomodified clay nanocomposite system was monitored by measuring the creep curves obtained while subjecting the sample to the combined action of temperature, tensile stress, and UV radiation. The creep curves of the irradiated samples were found to be lower than those of the non-irradiated ones and progressively diverging, because of the formation of branching and cross-linking due to photooxidation. This was further proved by the decrease of the melt index and the increase of the intrinsic viscosity; at the same time, the formation of carbonyl groups was observed. This behavior was more observable in the nanocomposite sample, because of its faster photooxidation kinetics.

## 1. Introduction

Polymer nanocomposites can show very interesting properties in comparison to the neat polymer matrix. It is well known that mechanical, rheological, and barrier properties can be significantly influenced by relatively low amounts of nanofiller [1,2,3,4,5,6].

On the other hand, creep behavior of nanocomposites has received less attention. Actually, only a few papers have focused on the creep behavior of polymer/clay nanocomposites. Most of them [7,8,9,10,11] focused on polyolefin-based nanocomposites (containing either clay or fumed silica) finding, on average, a significant increase of the creep resistance (provided that a good exfoliation degree is achieved, if clay was used); similar conclusions were found in a study [12] on elastomer-clay nanocomposites. In a previous work [13] we found that LDPE/clay nanocomposites, subjected to different temperatures and stresses, showed lower creep deformations in comparison to the neat polymer, and the relative differences increased upon increasing the applied stress and the temperature.

Therefore, it can be stated that the presence of nanoclay, on average, improves the creep resistance of polymer-based systems, mainly due to a higher rigidity.

On the other hand, Lv et al. [14] reported a completely different behavior on a PP/clay system, finding an increase of the deformation upon adding organomodified clay particles: they interpreted this behavior as invoking a poor dispersion of the nanoparticles and slippage phenomena of the non-exfoliated clay platelets; the addition of PP grafted with maleic anhydride, however, improved the creep resistance, because of the enhanced dispersion and intercalation of the clay particles.

Furthermore, no evidence exists of creep studies on LDPE nanocomposites with the simultaneous action of accelerated weathering. This is of particular importance in order to assess the behavior of such systems in real operating conditions under a constant stress and in the presence of sunlight (either outdoor or indoor). Usually, photooxidation of polymer systems is investigated by monitoring the changes in the chemical and molecular structures, morphology, and macroscopic properties after a given time of exposure to UV radiations. These measurements are usually performed upon modifying influencing physical parameters such as temperature, humidity, but not by applying a mechanical stress [15,16]. In a previous work, La Mantia et al. [17] presented a new equipment that is able to subject polymer samples to creep under different loads, but also to monitor the effect of the photooxidation due to temperature, humidity, and UV irradiation, thus fully simulating the real operating conditions for polymer-based items. In more detail, the changes of molecular weight and morphology, as well as the formation of new chemical groups, were taken into account by irradiating the specimens during a creep test and monitoring the subsequent deviations of their creep curves from those of the non-irradiated samples’ creep. It was found that the investigated systems (based on polyamide 6) underwent higher creep deformations upon being subjected to UV irradiation and this was attributed to the photooxidation, leading to a molecular weight reduction.

In a following work [18], La Mantia et al. investigated, with the same apparatus, the photooxidation behavior of a PP/organomodified clay nanocomposite sample, by monitoring the combined effects of temperature, tensile stress, and UV irradiation, finding a relationship between the time when the molecular weight started to decrease and the one when the creep curves of irradiated and non-irradiated samples started to diverge, with a more pronounced effect on the nanocomposites (if compared to the neat polymer matrix) due to their faster phohotoxidation kinetics.

Therefore, photoxidation can be monitored simultaneously to creep by simply monitoring the creep deformations in relationship with temperature, applied load, and humidity.

Since no systematic experimental studies are reported in the literature about the effect of the UV radiations on the creep behavior of LDPE-based nanocomposites, in this study we focused on investigating the combined creep–photooxidation behavior of LDPE/clay ones, by subjecting them to creep tests in presence of UV radiation and comparing them with the non-irradiated samples.

## 2. Materials and Methods

### 2.1. Materials and Sample Preparation

The materials used in this work were a film grade, low density polyethylene (LDPE) (Riblene MM20, Versalis, Italy) having a melt index of about 3.5 dg/min at 190 °C under 2.16 kg load, and a ditallowdimethylammonium modified montmorillonite (Dellite^®^ 72T, Laviosa Mineraria, Italy).

The nanocomposite sample (NPE) was obtained by compounding the LDPE with the organo-modified clay at 5 wt % concentration, in a corotating twin screw extruder (OMC, Saronno, Italy) with a temperature profile from 120 to 190 °C and a rotational speed of 180 rpm. Neat polymer was subjected to the same processing.

The specimens (70 mm × 13 mm × ≈ 0.5 mm) for the creep tests were obtained by cutting them off from compression molded sheets prepared by using a laboratory press, (Carver, Wabash, IN, USA) at 180 °C, with a pressure of approx. 7 bar and a compression time about 3 min. At least four specimens of each sample were tested.

### 2.2. Testing Equipment

Creep tests were performed at a temperature of 80 °C on both of the materials, by applying a stress of 1.5 MPa in a dedicated apparatus produced by IDEA (Termini Imerese, Italy), described in previous works [19,20,21]. Specifically regarding the UV source, it is obtained by using a chilled and filtered UV lamp (Osram UVA, 300 W, Munich, Germany), with the main wavelengths within the 350–2500 nm range and the measured photon flux (in the 280–320 nm wavelength range), about 348 mW/cm^2^, with the samples located at approx. 3 cm from the lamp. Reproducibility of the results was satisfactory (±4%).

### 2.3. Characterization

Differential scanning calorimetry (DSC) data were obtained using a DSC7 apparatus (PerkinElmer, Waltham, MA, USA), at a scanning rate of 10 °C/min in a temperature range from 30 to 210 °C. The value of the enthalpy of 100% crystalline LDPE, used for the determination of the crystalline degree, is 293 J/g.

X-ray diffraction measurements (XRD) were performed on a Empyrean system (PANalytical, Almelo, The Netherlands) equipped with a PIXcel1D detector. The Bragg–Brentano geometry comprises a Cu X-ray tube (operated at 40 KV and 30 mA; λ = 1.5418 Å). The patterns were collected in the 2θ range of 1–30°, the step size was 0.039°, and the exposure time was 240 s.

FT-IR ATR spectra were obtained by using a Spectrum One spectrometer (PerkinElmer, Waltham, MA, USA), using the Spectrum software, with a 4 cm^−1^ resolution. The oxidation path was monitored by calculating the absorbance ratio, obtained as the ratio between the band at 1720 cm^−1^ (–CO groups) and that at 2019 cm^−1^ (total carbon-hydrogen content) [18].

The change of the absorbance with the irradiation time was calculated as

(1) $$\mathrm{AC}=\frac{{\mathrm{Peak}}_{w,t}-{\mathrm{Peak}}_{w,0}}{{\mathrm{Peak}}_{w,0}}$$

where Peak_w,t_ is the value of the absorbance ratio of the investigated peak at a given time of photooxidation and Peak_w,0_ is the value of the same absorbance ratio for non-irradiated samples.

Melt Flow Index (MFI) was measured with a melt index apparatus (Ceast, Pianezza, Italy), at 190 °C under 2.16 kg load.

The intrinsic viscosity measurements, which directly provide information about the molecular weight, were obtained using a iVisc LMV 830 (Lauda, Lauda-Königshofen, Germany) capillary viscometer equipped with a Ubbelohde capillary viscometer, in an oil bath kept at 100 °C. The solution viscosity of each sample was determined as the average of five measurements. The samples were dissolved in boiling xylene at a concentration of 1 g/dL. All of the solutions were carefully filtered before the measurements.

The intrinsic viscosity values were thus calculated according to the Solomon-Ciuta equation

$$\left[\eta \right]=\frac{\sqrt{2}}{c}\;\sqrt{\eta_{\mathrm{sp}}-\ln\eta_{\mathrm{rel}}}$$

where c is the concentration of the polymer solution; η, η_sp_, and η_rel_ are, respectively, the intrinsic, specific, and relative viscosity.

The crosslinking degree of some materials was evaluated by calculating the gel percentage, which is directly correlated with it. This was obtained by performing solvent extraction tests in a Soxhlet apparatus, measuring the weight difference of the samples before and after 72 h extraction, and the calculating the ratio between the two measured weights (after/before).

## 3. Results and Discussion

**Figure 1:**
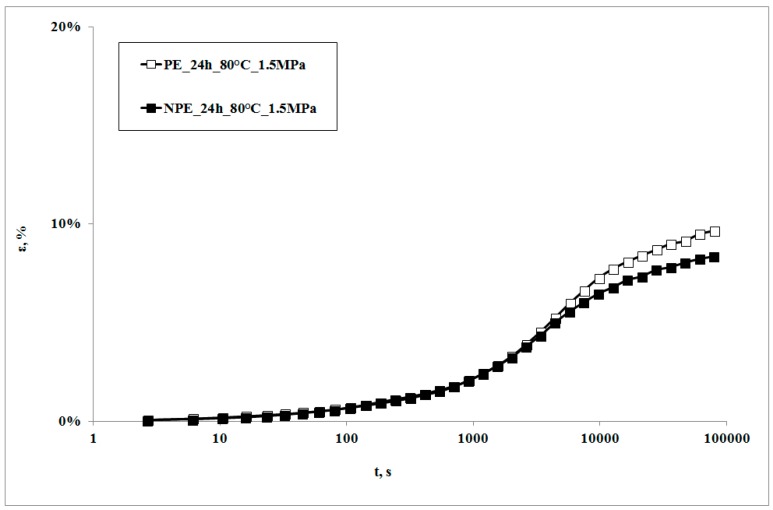
Creep curves at T = 80 °C.

**Figure 2:**
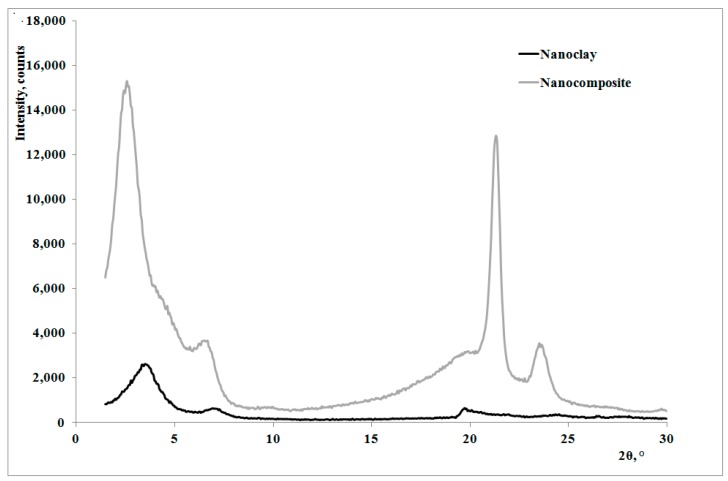
XRD traces of the nanoclay and the nanocomposite.

In Figure 1, creep curves of the neat polymer and the nanocomposite, subjected to the creep tests at 80 °C, are reported. The creep curve of the nanocomposite sample is almost superimposed on that of the matrix at lower creep times, while the nanocomposite shows lower deformations at higher creep times. This behavior can be correlated to the higher rigidity of the nanocomposite in comparison to the matrix [22,23,24], with a consequent lower deformability. This result is in agreement with that reported by other researchers on similar systems [7,10] and could be attributed to lower macromolecular mobility induced by the presence of clay particles and the obtained intercalation degree [13]: as shown by the XRD traces in Figure 2, the presence of the nanoclay in the nanocomposite shifts 2θ angle to lower values, and therefore the interplanar distance to higher ones, in particular from 2.62 to 3.44 nm, thus indicating that a moderate intercalation effect of the nanoclay, due to the LDPE chains , was obtained.

**Figure 3:**
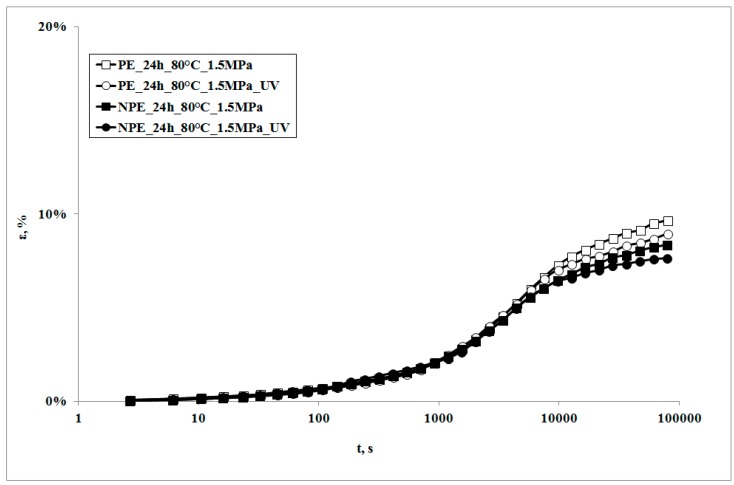
Creep curves of UV-irradiated and unirradiated samples, at *T* = 80 °C.

In Figure 3, creep curves of the two systems, subjected to the same stress, in presence of the UV irradiation or not, are reported.

It can be observed that the creep curves of the irradiated samples are almost superimposed on those of the non-irradiated samples at lower exposure times. On increasing the irradiation times, the deformability of the irradiated sample is slightly lower, but becomes even lower upon increasing the irradiation time. This behavior is the opposite to the one which was found in a polypropylene sample subjected to the same test [18]. Actually, in that sample, the creep curve of the non-irradiated sample was always below (i.e., showed lower values) the one of the irradiated sample. This behavior was attributed to the decrease of the molecular weight of the polymer matrix, due to the photooxidation undergone during the creep test: a lower molecular weight also causes a decrease of the viscosity, and thus an increase of the deformability of the material; this leads, in its turn, to an increase in the elongation under creep conditions.

The different creep response upon subjecting the samples to UV irradiation can be explained only by considering that some change in the molecular structure and/or the morphology must have occurred. This means that, in the case of LDPE matrix, a different photooxidation path must be considered as the deformability decreases. It is known that polyethylenes show, under UV irradiation, two contrasting degradation mechanisms, i.e., molecular weight reduction and branching and, eventually, cross-linking. Of course, both of the mechanisms occur at the same time and give rise to different final properties of the polymer. In particular, while the reduction of the molecular weight tends to increase creep deformation, the presence of branching and of cross-linking tends to mitigate it.

In Table 1, the melt flow index (MFI) of the samples (both irradiated and non-irradiated) at given exposure time is reported, as well as the intrinsic viscosity, gel fraction, and crystallinity degree.

These properties remain unchanged for all the non-irradiated samples of both LDPE and nanocomposite (data not reported here for sake of brevity), and this means that, over the investigated time frame, temperature and stress do not lead to significant degradation.

On the other hand, UV exposure causes significant changes in all of these characteristics, in both of the samples. In particular, the decrease of MFI and the increase of the intrinsic viscosity clearly indicate an increase of the molecular weight upon increasing the irradiation time. Unfortunately, with regard to the nanocomposite sample irradiated for 24 h, it was not possible to correctly measure the melt index and the intrinsic viscosity. With regard to the latter property, the first problems arose while preparing the solution, since the dissolution of the polymer sample was not complete; in other words, even after two hours at 100 °C, the solution was turbid and, after removing the flask from the heated plate, small gel residues precipitated. Furthermore, in any case, while pouring the solution in the capillary, some of the dissolved polymer precipitated along the capillary walls, during its normal flow from the flask into the capillary, regardless the time spent to perform this operation (i.e., precipitation was immediate). The residues formed during this stage (whose formation could not be avoided, as explained before) led to a decrease of the solution’s concentration and therefore to unreliable results, since the obtained intrinsic viscosity was way too low, if compared to all of the other investigated samples. For this reason, the value is not reported.

Furthermore, the previous considerations about the increase of the molecular weight upon increasing the irradiation time are also confirmed by the small amount of gel fraction found during the extraction tests of the above mentioned, most irradiated samples. This means that long chain branching formed during the irradiation [25] can evolve towards the formation of cross-linked structures. As a consequence of this dramatic change of the molecular structure, the MFI decreases and the viscosity increases. The increasing presence of carbonyl groups, finally, confirms the photooxidation undergone by the macromolecules.

All of the above described changes can be only due to the photo-oxidative degradation, caused by the UV radiations, which leads to a more viscous, more rigid structure and then to a less deformable material, as shown by the lower values of the creep curve.

It has been reported in our previous works [13,26] that the photooxidation of polyethylene leads to two competitive mechanisms, namely macromolecule breaking and the formation of branching and cross-linked structures. This means that, under the current testing conditions, the increase of molecular weight and the formation of branching are favored with respect to the breaking of the macromolecules. On increasing the exposure time, this photooxidation path leads to a small but significant formation of cross-linked structures. The polymers can be therefore represented by more rigid, higher molecular weight chains and rigid, insoluble, and infusible cross-linked structures floating in an ‘island’ of low viscosity, low molecular weight macromolecules. The decrease of the creep curves clearly suggests the predominant effect of branching and cross-linking on the creep behavior. Finally, the slight decrease of the crystallinity, which should lead to a more deformable material, is not able to contrast the increased rigidity of the photooxidized molecular structure. Creep tests during irradiation are able to correctly monitor the molecular changes achieved in the samples during the irradiation.

The creep curve of the irradiated nanocomposite sample is lower than that of the unirradiated sample and even lower with the increase of exposure time. It is worth noticing that the decrease of the creep curves in presence of UV irradiation is larger than that observed for the neat matrix in the same conditions.

All of the properties measured on this sample, Table 1, show the same trend of the pure matrix, but all of the values diverge much more from the virgin, unirradiated sample than those of the neat, unfilled matrix. This can be interpreted only by admitting that the photooxidative degradation undergone by the nanocomposite is more dramatic than that undergone by the neat matrix. This should mean that the degradation phenomena and their effects on the chemical and molecular structure, as well as on the morphology, are magnified in the presence of the nanoclay.

The faster photooxidation kinetics of the nanocomposite has been reported also in previous studies [16,26,27,28,29,30,31] and has been explained by demonstrating that the photodegradation, induced by the UV irradiation, is favored by the iron ions contained in the clay.

**Figure 4:**
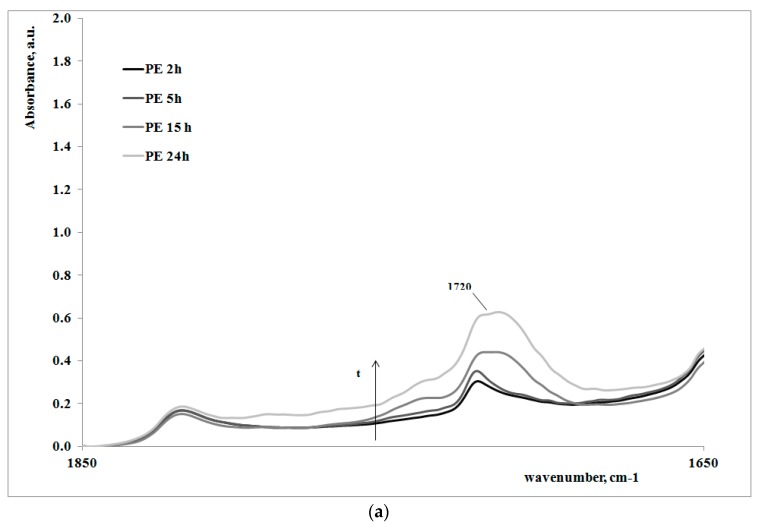

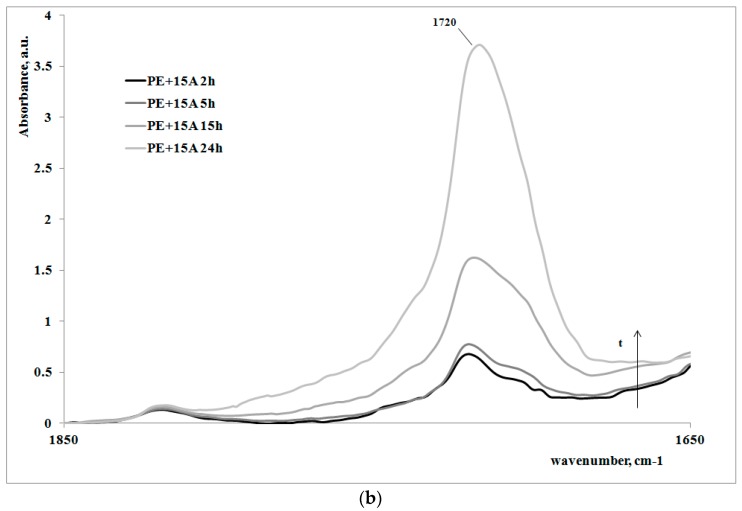
FTIR spectra of some irradiated and non-irradiated samples of polyethylene matrix (**a**) and nanocomposite (**b**).

In order to give a deeper analysis of the obtained results and further prove the correlation between creep behavior and photooxidation, FTIR spectra were measured on some irradiated samples at different exposure times. These are reported in Figure 4a,b, for the polymer matrix and the nanocomposite sample, respectively.

Of course, for both of the systems, on increasing the exposure time, the carbonyl band increases, clearly showing how the oxidation of the polyethylene macromolecules during the creep tests occurs; however, the FT-IR spectra of the non-irradiated samples (here not reported), do not show any presence of carbonyl groups. In Table 2, the values of absorbance ratios of the carbonyl region upon increasing the photooxidation time are reported.

In can be easily observed that the amount of carbonyl groups, formed as a consequence of the UV irradiation, is higher for the nanocomposite sample in comparison with the neat polyethylene matrix. This result is in complete agreement with the previously discussed results regarding the change of the molecular structure of the nanocomposite with respect to the matrix.

## 4. Conclusions

In this work, the combined effect of creep and photooxidation on LDPE/clay nanocomposites was systematically investigated by means of a new apparatus able to simultaneously monitor the effect of applied stress, temperature, and UV irradiation on the samples. It was found that the creep deformations of the UV-irradiated samples were lower than those of the non-irradiated ones, and the differences progressively increased on increasing the irradiation time. This was attributed to the formation of branching and cross-linking induced by the photooxidation and was further demonstrated by the decrease of the melt index and the increase of the intrinsic viscosity (which confirmed that branching and crosslinking phenomena prevailed over chain-scission). FT-IR analysis showed the increasing presence of carbonyl groups on increasing the irradiation time, due to the photoxidation effects, and this presence was higher in the nanocomposite sample, due to its faster photooxidation kinetics.

## Figures and Tables

**Table 1:** MFI, intrinsic viscosity, gel fraction, crystallinity degree, and absorbance ratio of irradiated samples at given exposure times for LDPE and nanocomposite samples.

**LDPE**				
**Time (h)**	**MFI (g/10 min)**	**Gel fraction (%)**	**Intrinsic viscosity (cm^3^/g)**	**Crystallinity degree (%)**
0	3.55 ± 0.18	0	101.6 ± 7.1	27 ± 0.8
2	2.85 ± 0.14	0	102.4 ± 7.2	27 ± 0.8
6	2.36 ± 0.12	0	106.9 ± 7.5	25 ± 0.7
15	0.96 ± 0.05	0	126.2 ± 8.8	24 ± 0.6
24	0.61 ± 0.03	1 ± 0.06	142.1 ± 9.9	23 ± 0.6
**Nanocomposite**				
**Time (h)**	**MFI (g/10 min)**	**Gel fraction (%)**	**Intrinsic viscosity (cm^3^/g)**	**Crystallinity degree (%)**
0	1.80 ± 0.09	0	106.3 ± 7.4	25 ± 0.7
2	1.38 ± 0.07	0	107.9 ± 7.6	25 ± 0.7
6	1.01 ± 0.05	0	123.6 ± 8.7	23 ± 0.6
15	0.21 ± 0.01	0	134.6 ± 9.4	22 ± 0.5
24	-	5.3 ± 0.24	-	22 ± 0.6

**Table 2:** Absorbance ratio, in the carbonyl region, of irradiated samples at given exposure times for LDPE and nanocomposite sample.

**LDPE**	
**Time (h)**	**Absorbance ratio, CO**
0	0
2	0
6	0.33
15	2.6
24	4.8
**Nanocomposite**	
**Time (h)**	**Absorbance ratio, CO**
0	0
2	0.16
6	0.31
15	4.6
24	6.2
